# Unraveling the Complexities of Myeloid-Derived Suppressor Cells in Inflammatory Bowel Disease

**DOI:** 10.3390/ijms26073291

**Published:** 2025-04-02

**Authors:** Yangzhuangzhuang Zhu, Siyan Cao

**Affiliations:** Division of Gastroenterology, Department of Medicine, Washington University School of Medicine, St. Louis, MO 63110, USA; yangzhuangzhuang@wustl.edu

**Keywords:** inflammatory bowel disease, myeloid-derived suppressor cells, immunomodulation, experimental colitis, colon cancer

## Abstract

Myeloid-derived suppressor cells (MDSCs) regulate immune responses in many pathological conditions, one of which is inflammatory bowel disease (IBD), an incurable chronic disorder of the digestive tract and beyond. The pathophysiology of IBD remains unclear, likely involving aberrant innate and adaptive immunity. Studies have reported altered population of MDSCs in patients with IBD. However, their distribution varies among patients and different preclinical models of IBD. The expansion and activation of MDSCs are likely driven by various stimuli during intestinal inflammation, but the in-depth mechanisms remain poorly understood. The role of MDSCs in the pathogenesis of IBD appears to be paradoxical. In addition to intestinal inflammation, suppressive MDSCs may promote colitis-to-colon cancer transition. In this Review, we summarize recent progresses on the features, activation, and roles of MDSCs in the development of IBD and IBD-associated colon cancer.

## 1. Introduction

The term myeloid-derived suppressor cells (MDSCs) was first proposed in 2007 to describe a heterogeneous group of immature myeloid cells with potent immunosuppressive ability [1]. MDSCs develop from a common myeloid progenitor in response to persistent stimulation from conditions including infection, inflammation, and cancers. MDSCs are divided into two major groups, monocytic MDSCs (M-MDSCs) and granulocytic/polymorphonuclear MDSCs (G-MDSCs/PMN-MDSCs). They share a similar phenotype and morphology with mature monocytes/neutrophils but possess distinct functional characteristics. The main role of MDSCs is believed to terminate persistent immune responses and avoid tissue damage. The initial study of MDSCs was carried out in cancers, where this activity of MDSCs was amplified by cancer cells for them to escape from immune surveillance and evade immunotherapy [2]. In recent studies, the accumulation of MDSCs was also observed in a variety of autoimmune and inflammatory diseases, such as rheumatoid arthritis, systemic lupus erythematosus, psoriasis, and inflammatory bowel disease (IBD). However, the role of MDSCs in those diseases remains poorly understood.

IBD, consisting of ulcerative colitis (UC) and Crohn’s disease (CD), is a chronic inflammatory disorder characterized by relapsing inflammation of the gastrointestinal tract and extraintestinal organs [3]. IBD affects broad populations regardless of age, sex, ethnicity, and region. The latest Global Burden of Disease Study reported approximately 4.9 million prevalent cases of IBD, with 40,998 deaths and 1.62 million disability-adjusted life-years (DALYs). China and the United States have the highest number of cases [4]. Despite its relatively low mortality, the chronic disease course and resulting healthcare needs have imposed a substantial economic burden on IBD patients and global healthcare systems [5]. Moreover, patients with IBD are at higher risk of developing colorectal cancer (CRC), which is responsible for 10% to 15% of deaths in IBD [6]. The pathogenesis of IBD remains unclear. Some evidence suggests that it results from a dysregulated and persistent inflammatory response in a susceptible host to commensal microbes and other environmental stimuli [7]. The dysfunction in innate and adaptive immune responses has been highlighted in IBD pathogenesis [8]. Recent studies offered insights into MDSCs as an important component in maintaining intestinal homeostasis. In this Review, we provide an overview of the recent studies on MDSCs in IBD and a summary of the features, expansion, activation, and roles of MDSCs in IBD and IBD-associated CRC.

## 2. Defining Features of MDSCs

M-MDSCs and G-MDSCs share a similar phenotype and morphology with mature monocytes and neutrophils, respectively. However, M-MDSCs and G-MDSCs exhibit their distinct transcriptional profile and functional characteristics.

### 2.1. Markers of Mouse MDSCs

MDSCs were originally described in tumor-bearing mice. Numerous phenotype markers were identified to define MDSCs and their subsets [9]. The typical phenotype of mouse MDSCs is CD11b^+^Gr-1^+^, which is almost universally discriminant in experimental models [10,11]. Given that CD11b^+^Gr-1^+^ cells also include monocytic, granulocytic, and immature myeloid cells, other markers such as CD80, CD115, and CD124 were proposed to describe suppressive MDSCs in some cancer models [12,13,14]. It is important to notice that those functional surface markers may not be discriminant in M-MDSCs and G-MDSCs in some experimental models [11]. The subpopulations G-MDSCs and M-MDSCs are generally identified based on the expression of two epitopes of Gr-1, Ly6G and Ly6C. The CD11b^+^Ly6C^lo/int^Ly6G^high^ cells with high side scatter (SSC) are identified as G-MDSCs, whereas CD11b^+^Ly6C^high^Ly6G^−^ cells with low SSC are identified as M-MDSCs. Functionally, M-MDSCs were thought to be more immune suppressive than G-MDSCs. To better distinguish the two subpopulations, CD49d has been suggested as an alternative marker for Gr-1 in tumor-bearing mice and an IBD mouse model. CD11b^+^CD49d^+^ cells expressing monocytic markers (e.g., F4/80) and Ly6G^−^Ly6C^high^ displayed a similar functional characteristic with M-MDSCs [15]. Future studies should characterize the subtypes of pathologically expanded myeloid cells in different preclinical IBD models.

In addition, morphologic analysis showed a multi-lobed or donut-shaped nucleus in those granulocytes, which may help distinguish G-MDSCs from neutrophils in mice [16]. In chronic colitis induced in immunodeficient *Rag*^−/−^ mice after transfer of naïve CD4^+^CD45RB^high^ T cells, approximately 75% of Gr-1^+^ cells in the spleen exhibited a donut-shaped nucleus characteristic of “bands” that represented newly released, partially matured neutrophils including G-MDSCs, whereas Gr-1^+^ cells isolated from colonic lamina propria (LP) were mostly mature “segmented” neutrophils [17].

### 2.2. Markers of Human MDSCs

Gr-1 is not expressed in human leukocytes, which makes identifying human MDSCs more complicated. Some studies identified human MDSCs as HLA-DR^−^CD11b^+^CD33^+^ cells, which are insufficient to characterize all MDSCs in humans. For MDSC subtypes, M-MDSCs are characterized by monocytic markers CD14^+^CD15^−^, but differ from monocytes by HLA-DR^−/lo^ expression. G-MDSCs resemble granulocytes and are characterized as CD14^−^CD15^+^ and CD66b^+^. Myeloid cell marker CD33 can be used to replace CD11b. M-MDSCs express CD33, while PMN-MDSCs are CD33^dim^ [18]. However, none of those markers is specific and they are unable to distinguish G-MDSCs and M-MDSCs from neutrophils and monocytes. It was reported that G-MDSCs had higher forward scatter (FSC) and similar SSC compared to neutrophils, suggesting that G-MDSCs are larger than neutrophils while maintaining similar granularity [19]. It is also possible to use gradient centrifugation (1.077 g/mL density for standard mononuclear cell isolation) to physically separate G-MDSCs from neutrophils, with G-MDSCs enriched in the low density. However, this approach is limited by neutrophil contamination in the low-density fraction. Furthermore, the expression of HLA-DR is not sufficient to differentiate M-MDSCs from monocytes in some circumstances when M-MDSCs differentiate into an antigen presenting cell-like phenotype [20]. Therefore, finding reliable characteristic markers of human MDSCs remains one of the most pressing issues in this field.

## 3. Inflammation-Driven MDSC Expansion and Activation in IBD

MDSCs likely arise from persistent stimulation of the myeloid cell compartment in the settings of chronic inflammation. An overlapped two-signal model was proposed to describe the expansion and activation of MDSCs [21]. The first signal for MDSC expansion is induced by myeloid growth factors and inflammatory stimuli, including granulocyte-macrophage colony-stimulating factor (GM-CSF), M-CSF, G-CSF, interleukin-6 (IL-6), etc., which activate the signal transducer and activator of transcription 3 (STAT3) and STAT5 in MDSCs. The second signal that manifests in promoting MDSCs’ immune suppressive activity is provided by toll-like receptor (TLR) ligands as well as proinflammatory cytokines including interferon-γ (IFN-γ), IL-1β, IL-13, etc., through nuclear factor kappa-B (NF-κB), STAT1, STAT6, and cyclo-oxygenase-2 (COX-2)/prostaglandin E2 (PGE2). Though a “one-signal” model implies that one factor is sufficient to promote the differentiation and activation of MDSCs, the “two-signal” model, in which the accumulation of suppressive MDSCs requires two strong signals, is more favored by current evidence [21]. This “two-signal” model initially explained how immature myeloid cells become immune-suppressive MDSCs in response to environmental clues.

### 3.1. MDSC Distribution in IBD Patients and Mouse Models of IBD

Substantial variability in the ratio of G-MDSCs/M-MDSCs has been reported in human diseases including IBD. M-MDSCs are considered the main subtype of MDSCs in IBD based on their increased numbers in the peripheral blood of patients with UC or CD [22,23,24]. Notably, the changes in MDSC frequencies were inconsistent in different studies. One study reported increased HLA-DR^−/lo^CD14^−^CD33^+^CD15^+^ cells, which can be classified as G-MDSCs, in patients with active CD or UC. However, no elevation of HLA-DR^−/lo^CD14^−^CD33^+^CD15^+^ MDSCs was found in patients with quiescent CD or UC. These increased G-MDSCs were shown to promote T cell proliferation in vitro [25]. Other studies identified increased suppressive CD14^+^HLA-DR^−/lo^ MDSCs (M-MDSCs) in the peripheral blood of patients with active CD and UC, with no change in G-MDSCs in the blood samples [22,23,24]. Moreover, the frequency of M-MDSCs in patients with active CD was higher than those with CD in remission, which remained higher than that in healthy individuals; the frequency of M-MDSCs in patients with active UC was also elevated than that in patients with quiescent UC [23]. Those studies suggest that the expansion of M-MDSCs and G-MDSCs may be related to the disease activity of IBD. The changing inflammatory environment during the progression of IBD and treatments may also alter the subtypes, function, and distribution of MDSCs. Further studies are needed to provide more detailed information on the distribution of both MDSC subtypes in the peripheral blood and intestinal tissues of IBD patients with different disease activity and therapeutic interventions.

Increased MDSCs were reported in the spleen, peripheral blood, bone marrow, mesenteric lymph nodes, Peyer’s patches, and colonic LP in some murine models of IBD (Table 1). Some researchers proposed that MDSCs expanded only in T cell-dependent colitis [22]. One study reported elevated MDSCs in 2,4,6-trinitrobenzene sulfonic acid (TNBS)-induced colitis, whereas no significant change was found in the spleen or mesenteric lymph nodes of dextran sulfate sodium (DSS)-induced acute colitis that does not require T or B lymphocytes [26]. However, more studies suggested that the accumulation of MDSCs is not unique to T cell-dependent colitis (Table 1). One of these studies showed increased MDSCs in the spleen and LP in TNBS-induced colitis, whereas only LP MDSCs were upregulated in DSS-induced acute colitis [27]. This was interpreted as the local inflamed colonic LP being the primary site where MDSCs are recruited. Experiments using colitis induced in immunodeficient mice may help address the requirement for T cells in regulating the numbers and activities of MDSCs. Mice’s genetic backgrounds, microbiome, and dosage/timing of DSS may also account for these different results in the literature.

### 3.2. Signaling Pathways Regulating MDSC Expansion in IBD

**Figure 1:**
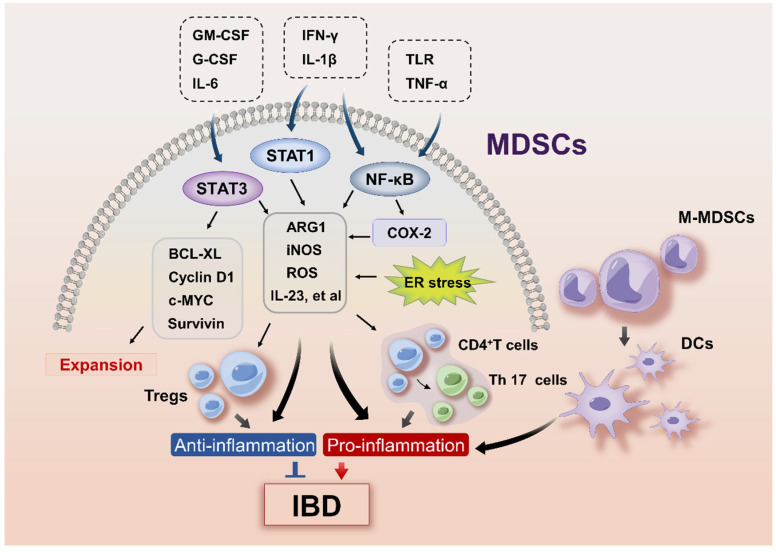
The paradoxical role of MDSCs in the pathogenesis of IBD. The expansion of MDSCs is driven by myeloid growth factors and inflammatory signals including GM-CSF, G-CSF, IL-6, etc. that activate STAT3. Activation of STAT3 in MDSCs upregulates their expression of BCL-XL, cyclin D1, c-MYC, and survivin protein to prevent apoptosis and promote proliferation of MDSCs. Inflammatory mediators including TLRs, IFN-γ, IL-1β, and TNF-α promote MDSCs activities by activating STAT3, STAT1, and NF-κB signaling pathways, leading to upregulation of arginase 1 (ARG1), inducible nitric oxide synthase (iNOS), and NADPH oxidase 2 (NOX2) that produces reactive oxygen species (ROS). These suppressive factors may alleviate IBD by suppressing effector T cell responses and promoting Treg activity. On the contrary, Th17 cells induced by MDSCs-derived IL-23 and DCs differentiated from M-MDSCs may exacerbate IBD.

The intracellular pathways reported to orchestrate MDSCs in IBD include STAT3 and GM-CSF (Figure 1). The upregulation of STAT3 is a hallmark of MDSCs and is directly involved in their expansion and activation [13]. The abnormal and persistent activation of STAT3 in MDSCs upregulated the expression of B-cell lymphoma XL (BCL-XL), cyclin D1, c-MYC, survivin protein, and S100 calcium-binding proteins A8/9 (S100A8/9) that prevented their apoptosis and promoted proliferation [13]. In recent years, studies have reported *Stat3*-activated suppressive MDSCs in IBD mouse models [31,32]. In a DSS-induced colitis model, the hyperactivation of *Stat3* in myeloid cells in *gp130757*^F/F^ mice ameliorated intestinal inflammation, which was attributed to the expansion and activation of MDSCs [36]. DSS-treated *LysM*cre/*Stat3*^flox^ mice with myeloid-specific *Stat3* deficiency showed diminished G-MDSCs and M-MDSCs in the colon compared to *gp130757*^F/F^ mice [36]. In addition, the STAT signals may control MDSC generation in mice. It was shown that the inhibition of enhancer of zest homolog 2 (*Ezh2*), a major histone methyltransferase, promoted the differentiation of hematopoietic progenitor cells (HPCs) into MDSCs by activating the Janus kinase (JAK)-STAT and TNF signaling pathways in DSS-induced colitis [33].

STAT3 activation in MDSCs may require growth factors including GM-CSF, G-CSF, and IL-6 [37]. GM-CSF and IL-6 are the major myeloid growth factors responsible for MDSC expansion, which was confirmed by a study showing that human MDSCs with potent suppressive capacity were best generated by GM-CSF plus IL-6, whereas GM-CSF alone had weaker effects [38]. The expansion of MDSCs in the colon of mice with colitis was accompanied by increased GM-CSF and IL-6 in different IBD mouse models [22,24,35], suggesting that GM-CSF and IL-6 may be involved in MDSC expansion in IBD. GM-CSF is mainly secreted by CD4^+^ T cells, group 3 innate lymphoid cells, and Paneth cells in the intestinal mucosa [39]. Notably, GM-CSF is also one of the signature cytokines for the IL-23 pathway that precipitates the onset and progression of IBD [40]. In addition to STATs, NOD-like receptor protein 3 (NLRP3) is implicated in the recruitment of MDSCs to the tumor site [41,42]. NLRP3 mediates the conversion of pro-caspase-1 to its mature form to generate IL-1β and IL-18 [43], while caspase-1-mediated inflammasome pathways control TNBS-induced colitis in mice [28]. MDSCs were significantly upregulated in the bone marrow, spleen, Peyer’s patches, and LP of the TNBS-treated WT mice but not in TNBS-treated caspase-1 knockout mice, suggesting that caspase-1-mediated inflammasome pathways promote the recruitment of MDSCs.

### 3.3. Signaling Pathways Involved in MDSC Activation in IBD

The STAT3 and NF-κB pathways appear to be the major regulators of MDSC activation in IBD (Figure 1). STAT3 and NF-κB upregulated the expression of arginase 1 (ARG1) and inducible nitric oxide synthase (iNOS), the main suppressors of MDSCs in murine colitis induced by dinitrobenzene sulfonic acid (DNB) or DSS [31,32]. NF-κB is positively related to the severity of intestinal inflammation [44] and is activated by various proinflammatory mediators, such as TLR ligands, IFN-γ, IL-1β, and TNF-α, all of which have been shown to increase the suppressive activity of MDSCs [21,45,46]. Indeed, increased concentrations of IL-1β, TNF-α, and IFN-γ in DSS colitis was accompanied by the expansion of functional MDSCs [47]. IFN-γ produced by T cells upregulated ARG1 and iNOS in MDSCs in a STAT1-dependent manner; while the blockade of IFN-γ abolished MDSC-mediated T cell suppression [48]. The enhanced IFN-γ response was also accompanied by upregulation of iNOS in MDSCs in *Il-17a*^−/−^ mice in *Il10*-deficiency-induced IBD [35]. In addition, the canonical activation of NF-κB leads to the production of COX-2 [49]. COX-2 and COX-2-derived PGE2 were shown to stimulate the expression of ARG1 and iNOS to activate suppressive MDSCs in cancers [50,51,52], although the findings have not yet been confirmed in IBD. In recent years, endoplasmic reticulum (ER) stress response has also emerged as an important mechanism regulating MDSC activation. Three major sensors of ER stress exist in mammalian cells: protein kinase RNA-like ER kinase (PERK), inositol-requiring enzyme 1 (IRE1), and activating transcription factor 6 (ATF6). CCAAT-enhancer-binding protein homologous protein (CHOP) and spliced X-box binding protein-1 (XBP1s) are vital effectors of the PERK and IRE1 pathways, respectively [53]. ER stress promotes the suppressive activity of MDSCs by inducing the expression of ARG1, iNOS, and NADPH oxidase 2 (NOX2) [54,55]. ER stress and the unfolded protein response (UPR) have been functionally and genetically linked to human IBD [56,57]. It remains elusive how ER stress in IBD regulates the expansion and activation of MDSCs.

### 3.4. Hormone-Driven MDSC Activity in IBD

In a DNB-induced IBD mouse model, estrogen steroid 17β-estradiol was reported to mitigate IBD by augmenting the suppressive activity of MDSCs [31]. In DSS colitis, the activation of estrogen receptors increased MDSC-derived ARG1 and consequently alleviated colitis [32]. The cases, deaths, and DALYs of IBD were higher among females than males [4]. Several studies have reported sex differences in patients with IBD. A pooled analysis of population-based studies from the Asia–Pacific region showed that CD and UC patients were predominantly male in most age groups from adolescence to middle/late-middle age [58]. However, a pooled analysis of population-based studies from Western countries reported that female patients had a lower risk of CD during childhood but a higher risk of CD thereafter, whereas men had a higher incidence of UC than women after the age of 45 [59]. A cross-sectional cohort study uncovered that over half of the women with IBD reported worsening symptoms during menses, whereas symptom worsening during pregnancy was more commonly seen in UC than CD. Moreover, oral contraceptive use has been associated with an increased risk of CD in female patients [60]. Sex differences in immune response are likely related to the differential organization of chromosomes, reproductive organs, and levels of sex steroids [61]. The distribution of MDSCs and subsets has been reported to be sex-specific in glioblastoma [62], systemic lupus erythematosus [63], obesity [55], arthritis [64], and breast milk for newborns [65]. The sex-dependent variations affect tumorigenesis and immunotherapy response [66]. Bayik, et al. reported that males had enhanced M-MDSCs accumulation in the tumor microenvironment, while females had elevated G-MDSCs in the peripheral circulation. This distinctive abundance of MDSC subsets may result in sex differences in anti-tumor immunity. It was further identified that G-MDSCs targeted with an anti-IL-1β antibody provided survival benefits to female tumor-bearing mice, while M-MDSCs targeted with fludarabine extended survival in male tumor-bearing mice [67]. These studies indicate that the unique characteristics of MDSC subsets driven by sex may determine their response to different therapies. Further investigations are needed to explore how sex is a factor in regulating MDSCs in IBD.

## 4. MDSCs in IBD Pathogenesis

MDSCs are known for their immunosuppressive activity on T cells, B cells, and natural killer (NK) cells. M-MDSCs and G-MDSCs utilize different mechanisms to interfere with the proliferation, trafficking, and viability of lymphocytes. G-MDSCs express high levels of ARG1 and NOX2, leading to increased reactive oxygen species (ROS) and decreased nitric oxide (NO) [13]. ROS is short-lived and unstable, which requires direct MDSC–T cell contact that renders T cells unresponsive to specific antigens. M-MDSCs appear to be more suppressive than G-MDSCs when assessed on a per-cell basis [68]. M-MDSCs expressed high levels of ARG1, transforming growth factor (TGF)-β, IL-10, and iNOS, which resulted in high NO and low ROS production [10,68,69,70]. NO has a longer half-life than ROS and allows cell–cell interaction via cellular proximity without direct cell contact. This enables M-MDSCs to suppress T cell responses both in antigen-specific and non-specific manners.

### 4.1. The Protective Role of MDSCs in IBD

The excessive and prolonged activation of adaptive immunity drives tissue damage in IBD. Adaptive responses are orchestrated by a combination of resident and recruited cell populations, including a variety of T cells including T-helper (Th)1, Th2, Th17, and regulatory T cells (Tregs) [7]. Most studies of MDSCs in IBD have concentrated on their interaction with T cells. MDSCs were shown to inhibit the proliferation of CD4^+^ T cells and promote the differentiation of Tregs and Th17 cells [71,72]. Touw, et al. reported that glatiramer acetate promoted MDSC-dependent Treg conversion while inhibiting the secretion of proinflammatory cytokines in DSS colitis in mice [73]. Lee, et al. showed that Tregs and MDSCs established a positive feedback loop during murine colitis, in which Treg enhanced the formation of M-MDSCs but not G-MDSCs, and boosted their inhibitory functions by secreting TGF-β [74].

The protective role of MDSCs was first uncovered by cell transfer and depletion experiments. Most studies confirmed the beneficial effects of MDSC transfer in mouse IBD models. The adoptive transfer of splenic MDSCs isolated from mice with colitis attenuated both acute and chronic intestinal inflammation [22,75]. The depletion of myeloid cells using an anti-Gr-1 antibody exacerbated intestinal inflammation in mice [76,77]. ARG1, iNOS, and ROS appear to be the major factors that mediate the suppressive activity of MDSCs on T cells in IBD. MDSCs inhibited T cell proliferation through competition for L-arginine, which serves as a substrate for iNOS and arginase [78,79]. A similar expression of ARG1 was found in CD14^+^CD19^−^HLA-DR^−/lo^ M-MDSCs from healthy controls and IBD patients. ARG-1-expressing M-MDSCs suppressed the proliferation of patients’ PBMCs in a dose-dependent manner [22]. In DSS colitis, *Arg-1* deletion in myeloid cells attenuated the protective role of MDSCs during colitis by upregulating IL-17A and IL-17F, while the adoptive transfer of suppressive MDSCs alleviated colitis in *Arg^myeKO^* mice [32].

ROS has cytotoxic effects in many conditions but is intrinsically involved in the transcriptional and metabolic reprogramming of MDSCs, thus influencing their differentiation and immunosuppressive activity. MDSC-derived ROS modified TCR and CD8 molecules and caused CD8^+^ T cells to lose their ability to bind phosphorylated MHC, leading to antigen-specific tolerance [80]. iNOS interplayed with ARG1 and NOX-derived ROS to produce peroxynitrite (ONOO-) that inhibits the proliferation, migration, and antigen-specific non-responsiveness of T cells [81,82,83]. IFN-γ produced by activated T cells triggered iNOS expression in suppressive MDSCs [84]. In a *Rag1^−/−^* IBD model, expanded M-MDSCs suppressed T cell proliferation in an iNOS-, IFN-γ-, COX-1/2-, and cell contact-dependent manner, without inducing their apoptosis [34]. M-MDSCs suppressed the production of Th1- and Th2-type cytokines and enhanced the generation of Th17 and foxp3^+^ T cells [34]. In addition, the neuroendocrine pathways also participate in the regulation of MDSC activity. Zheng, et al. reported an IL-10-dependent inhibitory effect of M-MDSCs on DSS-induced colitis. Acetylcholine promoted IL-10 secretion by M-MDSCs and suppressed inflammation by activating the nicotinic acetylcholine receptor (nAChR)/extracellular signal-regulated kinase (ERK) pathways, whereas the expression of iNOS and ARG1 remained unchanged [85]. Pituitary hormone α-MSH was found to act on bone marrow progenitors to boost myelopoiesis, MDSC accumulation, and immunosuppression through its melanocortin receptor MC5R [86]. The finding that MC5R displayed cytoplasmic staining at the mononuclear and polymorphonuclear inflammatory infiltrate levels in IBD patients suggests a potential role of melanocortin system in regulating MDSCs in IBD [87].

### 4.2. The Proinflammatory Function of MDSCs in IBD

Although previous findings suggested a protective role of MDSCs in IBD, several studies showed a proinflammatory role of MDSCs in intestinal inflammation (Figure 1). CD33^+^CD15^+^HLA-DR^−/lo^CD14^−^ G-MDSCs isolated from peripheral blood of patients with active IBD was found to stimulate CD4^+^CD25^−^ T cell proliferation [25]. Furthermore, the adoptive transfer of bone marrow-derived Gr1^+^CD11b^+^ MDSCs to TNBS-treated mice worsened the colitis, suggesting that this inflammatory environment switched off the suppressive capacity of MDSCs. The proinflammatory role of MDSCs has been reported in multiple autoimmune diseases, such as psoriasis, systemic lupus erythematosus, and rheumatoid arthritis [88,89,90]. The excessive proliferation of MDSCs led to augmented Th17 cell infiltration through the secretion of inflammatory cytokines including IL-23, IL-1β, and IL-6 [91,92].

The underlying mechanisms for the paradoxical effects of MDSCs in IBD remain unclear, one explanation being related to iNOS and ROS. iNOS enhances production of NO, which plays a complex role in the pathogenesis of IBD. NO compromises the integrity of intestinal epithelial barrier, which allows the translocation of luminal bacteria, toxins, and antigens into the mucosa, initiating intestinal inflammation [93]. Increased NO produced by MDSCs was shown to exacerbate colitis and promote death of *Il-10^−/−^* mice with colitis, though those MDSCs remained suppressive to CD4^+^ T cells [35]. In addition, Zigmond, et al. reported proinflammatory CX3CR1-GFP^int^Ly6C^hi^ monocytes, with a similar phenotype as M-MDSCs, highly expressed *Trem*, *Inos*, *Il-6*, and *Il-23* in mice with DSS colitis. The formation of those proinflammatory monocytes required TLR2 and nucleotide-binding oligomerization domain 2 (NOD2) [20]. ROS may also contribute to the pathogenic function of MDSCs in IBD by causing progressive cellular damage and tissue destruction [80].

### 4.3. Plasticity of M-MDSCs in IBD

In recent years, more evidence points out the functional plasticity of heterogeneous MDSCs in experimental models of IBD. These characteristics are mainly associated with M-MDSCs, whereas G-MDSCs are non-proliferating cells with a short half-life [94]. Several studies uncovered the possible differentiation fates of Ly6C^hi^ monocytes and identified them as a driver for colonel inflammation in IBD [20,95,96]. The Ly6C^hi^ monocytes have a similar phenotype to M-MDSCs in IBD. Chen, et al. reported that CD11b^+^CD14^+^CX3CR1^+^ dendritic cells (DCs) in the intestinal LP were derived from Ly6C^hi^ monocytes in a GM-CSF-dependent manner. These Ly6C^hi^ monocytes-derived DCs exacerbated DSS-induced colitis by secreting TNF-α [95]. Aymeric, et al. further elaborated the distinctive patterns of M-MDSCs in the steady state and inflammatory settings. Ly6C^hi^ monocytes in the blood were identified as the precursors of F4/80^hi^CX3CR1^hi^CD11c^+^ macrophages in noninflammatory environment, whereas Ly6C^hi^ monocytes were recruited to the colonic LP and differentiated into proinflammatory CD103^−^CX3CR1^int^CD11b^+^ DCs in a T cell transfer model of colitis. Those proinflammatory DCs produced high levels of IL-12, IL-23, iNOS, and TNF-α and drove the differentiation of IFN-γ–producing T cells [96]. Zigmond, et al. further uncovered the differentiation of Ly6C^hi^ monocytes with time in murine IBD: grafted Ly6C^hi^ monocytes differentiated into CX3CR1-GFP^int^Ly6C^hi^F4/80^lo^CD11c^−^ cells 1 day after adoptive transfer into DSS-challenged mice, then, by 3 days after, they became antigen-presenting CX3CR1-GFP^int^Ly6C^lo^F4/80^lo^ cells with high expression of MHCII and CD11c [20].

## 5. Effect of MDSCs During the Development of IBD-Associated Colorectal Cancer

**Figure 2:**
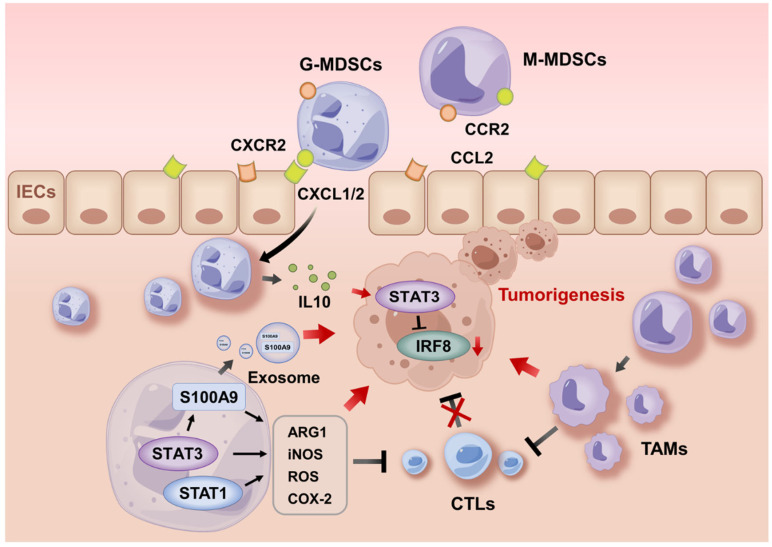
MDSCs in IBD-associated cancer. The upregulated expression of CXC chemokine receptor 2 (CXCR2) ligands and C-C motif chemokine ligand 2 (CCL2) in inflamed colonic mucosa and tumors recruited MDSCs via the ligand–receptor combination. MDSCs mediated the immune escape of tumor cells through both T cell-dependent and T cell-independent effects. IL-6, CCL2, G-CSF, and GM-CSF fostered MDSC accumulation and enhanced the suppression activities of MDSCs in a STAT3- and STAT1-dependent manner. ROS, ARG1, iNOS, and COX-2 in MDSCs promoted tumorigenesis by inhibiting cytotoxic T lymphocytes (CTLs). S100A9 in G-MDSC-derived exosomes increased the stemness of colon cancer cells, enhancing their susceptibility to CAC. IL-10 secreted by MDSCs activated STAT3 in colon epithelial cells, resulting in the silencing of tumor suppressor interferon regulatory factor 8 (IRF8). In addition, the plasticity of M-MDSCs may allow them to differentiate into M2-like tumor-associated macrophages (TAMs) that accelerate tumor progression.

Long-standing inflammation in IBD poses an increased risk of colorectal cancer [95]. MDSCs may contribute to IBD-initiated carcinogenesis by inhibiting cytotoxic T lymphocytes (CTLs), driving STAT3-dependent proliferation signals in intestinal epithelial cells (IECs), and inducing DNA damage by producing ROS [96]. Recent studies have highlighted the role of MDSCs in the immune microenvironment of IBD (Figure 2; discussion above). Poh, et al. showed that the chronic circulation of MUC1loCD11b+Gr1+ cells in the peripheral blood of IBD patients activated pro-tumorigenic pathways for colorectal cancer [97]. Mucin 1 (MUC1) is a tumor-associated antigen and a susceptibility gene for CD [98]. Low MUC1 expression on MDSCs promoted the expansion and suppressive capacity of MDSCs. This was accompanied by significantly reduced inflammatory lesions in the colon of mice with colitis-associated cancer (CAC) induced by azoxymethane (AOM)/DSS, indicating that the recruitment and sustained activation of MDSCs contribute to CAC [97].

### 5.1. MDSC Recruitment During Tumorigenesis

How are MDSCs recruited from the peripheral blood into inflamed colonic mucosa and tumors? Katoh, et al. showed that the pro-tumorigenic chemokine receptor CXCR2 was required for the recruitment of suppressive MDSCs, which was promoted by the PGE2-induced upregulation of CXCR2 ligands in colonic mucosa and tumors in an AOM/DSS-induced CAC model [97]. This was verified by the findings that downregulated CXCL2 reduced the infiltration of MDSCs and tumor growth, while early-life microbiota restricted MDSC-driven carcinogenesis by dampening the production of CXCL2 [98,99]. MDSC recruitment may also be mediated by other chemokines, cytokines, and growth factors. In AOM/DSS-induced and *T-bet^−/−^*, *Rag2^−/−^* CAC models, IL-6, CCL2, G-CSF, and GM-CSF (encoded by *Csf2*) fostered MDSC accumulation in evolving colonic tumors and boosted T cell suppression by MDSCs in a STAT-dependent manner [100,101,102]. In AOM/DSS-induced CAC, the number of MDSCs decreased significantly in *Csf2^−/−^* mice with thwarted cancer, whereas the adoptive transfer of MDSCs from tumor-bearing mice into *Csf2^−/−^* counterparts led to cancer recurrence [103]. Plasmacytoid dendritic cells (pDCs) impeded MDSC recruitment during AOM/DSS-induced CAC by producing type I IFN that suppresses the release of CCL2 and CXCL1 [104]. STAT1 is also required for G-MDSC accumulation in both the spleen and blood via IL-17 during the initial phase of CAC. The blockade of IL-17 in *Stat1*^−/−^ mice diminished G-MDSCs in murine CAC [105], indicating the functional role of STAT1 and STAT3 in the recruitment and activation of MDSCs.

Additionally, Chen, et al. reported that *Olfm4* deficiency in myeloid cells abrogated the recruitment of G-MDSCs, leading to delayed progression of AOM/DSS-induced CAC [106]. *Olfm4* deletion in myeloid cells blunted tumorigenesis but aggravated DSS-induced acute colitis in mice, which may be due to decreased frequency and immunosuppressive activity of G-MDSCs [106]. Those studies highlight a complex role of MDSCs in IBD and CAC: on one hand, suppressive MDSCs may alleviate intestinal inflammation, which is supported by data from adoptive transfer of suppressive MDSCs into IBD mice; on the other hand, expanded suppressive MDSCs in the peripheral blood may help initiate CAC by hampering anti-tumor immunity.

### 5.2. Activities of MDSCs During Tumor Progression

Recruited MDSCs mediate the immune escape of tumor cells through both T cell-dependent and -independent effects. Wang, et al. reported that increased commensal fungus in *Card^−/−^* mice induced MDSC accumulation, upregulated *S100a9* and *Arg-1* expression, and inhibited effector T cells, leading to accelerated AOM/DSS CAC in mice [107]. Wu, et al. further reported that the inhibition of S100A9 and STAT3 lowered ROS and ARG1 in MDSCs, restoring T cell responses in murine CAC [108]. In terms of non-T cell-dependent effects, Wan, et al. reported that S100A9 in G-MDSC-derived exosomes promoted the stemness of colon cancer cells, fostering their susceptibility to both AOM/DSS CAC and CT26 cell-induced cancer independent of T cells [109]. MDSC-derived IL-10 activated STAT3 in colonic epithelial cells that directly bond to the *Dnmt1* and *Dnmt3b* promoters to stimulate their expression, which then silenced tumor suppressor *Irf8* and advanced AOM/DSS CAC [110]. Moreover, the plasticity of M-MDSCs allowed them to differentiate into M2-like tumor-associated macrophages (TAMs) to drive tumor progression. Xun, et al. reported that natural product Dioscin halted AOM/DSS CAC by inducing the differentiation of MDSCs into the M1- but not M2-like phenotype [111]. G-MDSCs were reported to foster M-MDSC differentiation by producing exosomes. Starting from the inflammation stage of the AOM/DSS model, G-MDSCs accelerated the transition from colitis to cancer by secreting exosomes that drive the accumulation of M2 macrophages in colorectal tissue. G-MDSC-derived exosomes contributed to the differentiation of CD11b^+^Ly6G^−^Ly6C^+^ M-MDSCs into CD11b^+^F4/80^+^CD206^+^Ly6C^lo^ M2 macrophages through miR-93–5p cargo-mediated STAT3 inhibition [112].

Several other mechanisms in the activation of suppressive MDSCs may also regulate tumorigenesis. An elevated level of COX-2 was reported in AOM/DSS-induced murine CAC [113,114]. COX-2 produced by MDSCs may induce chromosomal instability and epithelial cell transformation leading to carcinogenesis [115,116]. Of note, COX-2 inhibitor Celebrex is approved by the U.S. Food and Drug Administration for the chemoprevention of familial adenomatous polyposis, an inherited cancer syndrome [117,118]. Excessive ROS production is associated with epithelial cell damage in IBD, and elevated ROS was observed in many types of cancers and likely involved in metastasis [119]. During IBD, MDSC-derived ROS may contribute to the development of dysplastic lesions [120,121]. In AOM/DSS-induced CAC, MDSCs displayed immunosuppressive activity by promoting STAT3-mediated ROS production [122]. Increased ROS generated by MDSCs may also induce ER stress, which plays a critical role in maintaining the suppressive function of MDSCs (as above). Suppressive MDSCs from cancer patients and from mice bearing EL4 and EG7 thymomas, LLC lung carcinoma, CT26 colon carcinoma, or 4T1 mammary carcinoma exhibited higher ER stress response compared with healthy controls. Upregulated XBP1 and CHOP may increase the expression of death receptor 5 (DR5), which shortened MDSCs’ lifespan to stimulate the proliferation of their precursors, thus supporting their expansion and accumulation [123]. The endogenous production of ROS or peroxynitrite in MDSCs has been shown to activate the expression of CHOP [124]. In tumor-bearing mice, *Chop* deficiency reduced the expansion and immunosuppressive activity of MDSCs [124]. Despite those findings, whether ER stress promotes colitis-associated tumorigenic process by inducing immunosuppressive MDSCs remains unknown.

## 6. Discussion

MDSCs expand systemically and locally as a part of the immunosuppressive response to limit tissue injury in IBD. MDSC subsets exhibit a broad distribution and expansion patterns across the disease stages of IBD (Table 1). The immunosuppressive function of MDSCs failed to dampen acute intestinal inflammation in multiple preclinical models. Moreover, persistent activation driven by recurrent epithelial injury or microbial translocation in IBD may reshape them into more heterogeneous phenotypes. Key unanswered questions include how MDSCs interact with gut microbiota, epithelial cells, and other immune cells to exhibit disease stage- and tissue-specific behaviors in IBD. Exploring the distribution and corresponding functions of MDSCs and their subsets in active disease and the remission of IBD will help better understand their clinical significance. In addition, the importance of physical conditions and functionality should not be underestimated in the management of IBD. E.g., physical activities were reported to be beneficial in quiescent or mild IBD and associated with a lower risk of active disease [125]. Recent studies introduced physical exercise as a promising strategy in cancers and inflammatory diseases due to its effect on immune cells, including inhibiting MDSC infiltration [126,127,128,129]. Whether physical exercise benefits IBD through regulating MDSCs remains unclear.

M-MDSCs were reported to be the major subset in peripheral blood in some IBD models [30,32,36,85]. There is substantial variability in M-MDSC/G-MDSC ratios and functions in the colon and peripheral lymphoid organs in mice. The mice’s genetic backgrounds, microbiome, and experimental design may count for these discrepancies. Though MDSCs were identified in the colon of almost all IBD mouse models and the peripheral blood of IBD patients, the reporting of colonic MDSCs in IBD patients has been limited. The discrepancy may be partly due to the lack of characteristic markers in humans, tissue-specific distribution of MDSCs, and stages of the disease. Previous reports have linked MDSC expansion to tumorigenesis. The immunostaining of UC colon tissues showed a low expression of the G-MDSCs marker CD15, which increased during the colitis-to-cancer transition [130]. The migration of MDSCs to tumors likely creates fertile ground for carcinogenesis and metastasis. The spleen has been reported to be the reservoir of CD11b^+^ Ly-6C^hi^ monocytic and CD11b^+^ Ly-6G^hi^ granulocytes in mice [131]. After migrating to the tumor site, the inflammatory and hypoxic tumor microenvironment upregulated the expression of suppressive factors in MDSCs and mediated the differentiation of M-MDSCs into TAM. Consistently, tumor MDSCs possessed a more potent suppressive activity than splenic MDSCs [132]. This may be partly explained by different G-MDSC/M-MDSC ratios in peripheral lymphoid organs and tumors. In the peripheral lymphoid organs of most cancer patients, G-MDSCs appear to be the major subset, whereas the proportion of M-MDSCs was higher in the tumor itself [133]. Further understanding of the mechanisms that drive the distribution of MDSCs and the shift between proinflammatory and anti-inflammatory phenotypes may help develop new immunotherapeutic interventions.

The therapeutic strategies to target MDSCs include chemotherapy, natural products, small molecule inhibitors and agonists, and antibodies, as well as the transfer of MDSCs or MDSC-derived exosomes. In cancers, chemotherapy drugs, antibodies, and inhibitors were tested to impede the recruitment and activity of MDSCs [134,135]. In IBD, targeted therapies including biologics and small molecules have revolutionized its management. TNF antibodies, IL-12/IL-23p40 antibody (ustekinumab), selective IL-23p19 antibodies (e.g., risankizumab, guselkumab, mirikizumab), and JAK inhibitors (e.g., tofacitinib, upadacitinib), which have demonstrated efficacy in IBD, may also impact myeloid cells [136]. For instance, tofacitinib was shown to induce significantly higher numbers of MDSCs and PMN-MDSCs in mice, accompanied by decreased Th17 cells and ILC1s [137,138]. Myeloid cells expressing Fc γ receptor 1 (or CD64) have been identified as a key source of IL-23 in inflamed IBD tissues. Guselkumab with a native Fc domain binds to CD64 on IL-23–producing inflammatory monocytes while capturing secreted IL-23 molecules [139], although the impact of guselkumab on MDSCs remains unknown. More preclinical and clinical studies are needed to define the role of MDSCs in response to those targeted treatments. Although the expansion of proinflammatory MDSCs was reported in patients and mice with IBD [20,25,35], clinical benefits of modulating MDSCs have not been reported. This may be explained by findings that the depletion of myeloid cells using anti-Gr-1 antibodies exacerbated intestinal inflammation. In addition, considering the critical role of suppressive MDSCs in carcinogenesis, the safety of MDSC activators or transfer of MDSCs needs to be carefully evaluated.

## 7. Conclusions

The expansion of MDSCs has been widely reported in IBD patients and mouse models of IBD. There is substantial variability in the activity and distribution of MDSCs in different models. The chronic inflammatory environment and sex hormones likely drive the expansion and activation of MDSCs. The functional and clinical significance of MDSCs in IBD have been clouded by both pro-homeostatic and proinflammatory roles. Moreover, prolonged suppressive G-MDSCs in the peripheral blood may promote the colitis-to-cancer transition in the colon. More studies are needed to explore the complex immune regulation of MDSCs in IBD and CAC in animal models and patients.

## Figures and Tables

**Table 1:** Distribution and frequencies of MDSCs in IBD mouse models.

Mice	Sex	Colitis Model	Colitis Stage	Distribution and Frequency											References
				PB	SP	MLN	LP	PP	BM	BaM	Int.	Col.	Ileum	IELs	
Balb/c	F	Intrarectal injection of 2–3% TNBS	Acute	-	Inc.	Inc.	-	-	-	-	-	-	-	-	[25]
C57BL/6	F	4% DSS in drinking water for 7 days	Acute	-	Inc.	-	Inc.	Inc.	-	Dec.	-	-	-	-	[28]
		2% DSS in drinking water for 5 days, drinking water for 5 days, 3 cycles	Chronic	-	Inc.	-	ns	Inc.	-	ns	-	-	-	-	
		Intrarectal injection of 2% TNBS	Acute	-	Inc.		Inc.	Inc.	-	Inc.	-	-	-	-	
Balb/c	-	DSS (5%, 7%, 7%) to the drinking water on days 0, 15, and 27, respectively, for 5 days	-	-	ns	ns	-	-	-	-	-	-	-	-	[22]
		Adoptive transfer of CD8+Tcells isolated from CL4-TCR mice into VILLIN-HA mice	Acute	-	Inc.	-	-	-	-	-	Inc.	-	-	-	
		SCID mice transferred with WT CD4^+^CD45RB^high^ T cells	Chronic	-	Inc.	-	-	-	-	-	-	-	-	-	
Balb/c	M	5% DSS induced in drinking water for 22 days	Chronic	Inc.	Inc.	-	-	-	Inc.	-	-	-	-	-	[23]
Balb/c	F	Intrarectally administered TNBS (1.5 mg and 1.8 mg) at a 1-week interval.	Acute	-	Inc.	-	Inc.	-	-	-	-	-	-	-	[27]
C57BL/6		4% DSS induced in drinking water for 5 days	Acute	-	ns	-	Inc.	-	-	-	-	-	-	-	
C57BL/6	M	2% DSS in drinking water for 7 days	Acute	-	Inc.	-	-	-	Inc.	-	-	Inc.	-	-	[29]
-	-	*Rag2*^−/−^ mice infected with *Helicobacter hepaticus*	Chronic	-	Inc.	-	Inc.	-	-	-	-	-	-	-	[30]
C57BL/6	F	4% 2,4-Dinitrobenzenesulfonic acid (DNB) enema for 5 days	Chronic	Inc.	Inc.	Inc.	Inc.	-	Inc.	-	-	-	-	-	[31]
C57BL/6	F	3.5% DSS in drinking water for 9 days	Acute	Inc.	ns	-	-	-	-	-	-	-	-	-	[32]
C57BL/6	F	2.5% DSS in drinking water for 7 days	Acute	Inc.	-	-	Inc.	-	Inc.	-	-	-	-	-	[33]
C57BL/6	-	*Rag2*^−/−^ mice transferred with WT CD4^+^CD45RB^high^ T cells	Chronic	Inc.	-	Inc.	Inc.	-	-	-	-	-	-	-	[34]
	-	3%DSS drinking in water for 7 days	Acute	-	Inc.	-	-	-	-	-	-	Inc.	-	-	
	F, M	*Tnf^ΔARE^* mice	Chronic	-	Inc.	-	-	-	-	-	-	-	Inc.	-	
C57BL/6	-	*Il10^−/−^* mice	Chronic	ns	ns	Inc.	Inc.	-	ns	-	-	-	-	-	[35]
C57BL/6	-	3% DSS in drinking water for 7 days	Acute	-	ns	ns	Inc.	-	-	-	-	Inc.	-	Inc.	[36]

Notes: F, female; M, male; Inc., increased; Dec., decreased; ns, not significant; PB, peripheral blood; SP, spleen; MLN, mesenteric lymph nodes; LP, lamina propria; PP, Peyer’s patch; BM, bone marrow; BaM, basal membrane; Int., small intestine; Col., colon; IELs, intraepithelial lymphocytes.

## Data Availability

No new data were created or analyzed in this study. Data sharing is not applicable to this article.

## Abbreviations

AOM, azoxymethane; ARG1, Arginase 1; ATF6, activating transcription factor 6; BCL-XL, B-cell lymphoma XL; CAC, colitis-associated cancer; CCL2, C-C motif chemokine ligand 2; CD, Crohn’s disease; CHOP, CCAAT-enhancer-binding protein homologous protein; COX-2, cyclo-oxygenase-2; CTLs, cytotoxic T lymphocytes; CRC, colorectal cancer; CXCR2, CXC chemokine receptor 2; DALYs, disability-adjusted life-years; DCs, dendritic cells; DR5, death receptor 5; DSS, dextran sulfate sodium; ER, endoplasmic reticulum; ERK, extracellular regulated protein kinases; Ezh2, zest homolog 2; FSC, forward scatter; GM-CSF, granulocyte-macrophage colony-stimulating factor; G-MDSC/PMN-MDSCs, granulocytic/polymorphonuclear MDSCs; HPCs, hematopoietic progenitor cells; IBD, inflammatory bowel disease; IECs, intestinal epithelial cells; IFN-γ, interferon-γ; IL-6, interleukin-6; IRE1, inositol-requiring enzyme 1; IRF8, interferon regulatory factor 8; iNOS, inducible nitric oxide synthase; JAK, Janus kinase; MDSCs, myeloid-derived suppressor cells; MUC1, mucin 1; M-MDSCs, monocytic MDSCs; NF-κB, nuclear factor kappa-B; NK cells, natural killer cells; NLRP3, NOD-like receptor protein 3; NO, nitric oxide; NOD2, nucleotide-binding oligomerization domain 2; NOX2, NADPH oxidase 2; ONOO-, peroxynitrite; PERK, protein kinase RNA-like ER kinase; PGE2, prostaglandin E2; ROS, reactive oxygen species; SSC, side scatter; ROS, reactive oxygen species; STAT3, signal transducer and activator of transcription 3; S100A8/9, S100 calcium-binding proteins A8/9; TAMs, tumor-associated macrophages; TGF-β, transforming growth factor-β; TLRs, toll-like receptors; TNBS, 2,4,6-trinitrobenzene sulfonic acid; Th1, T-helper 1; Tregs, regulatory T cells; UC, ulcerative colitis; UPR, unfolded protein response; XBP1, X-box binding protein-1; nAChRs, nicotinic acetylcholine receptors.
